# Supplementary material for: Regulation of Stanniocalcin Secretion by Calcium and PTHrP in Gilthead Seabream (Sparus aurata)
Source: Biology (Basel). 2022 Jun 4;11(6):863. doi: 10.3390/biology11060863 (PMC9219694; doi:10.3390/biology11060863)

Regulation of Stanniocalcin secretion by calcium and PTHrP in gilthead seabream (*Sparus aurata*)

Ignacio Ruiz-Jarabo, Silvia F. Gregório and Juan Fuentes

SUPPLEMENTARY MATERIAL

Figure S1. Corpuscles of Stannius in gilthead seabream. Photograph of the location of the two Stannius corpuscles (red arrows) in the caudal region of the kidney in *Sparus aurata*.

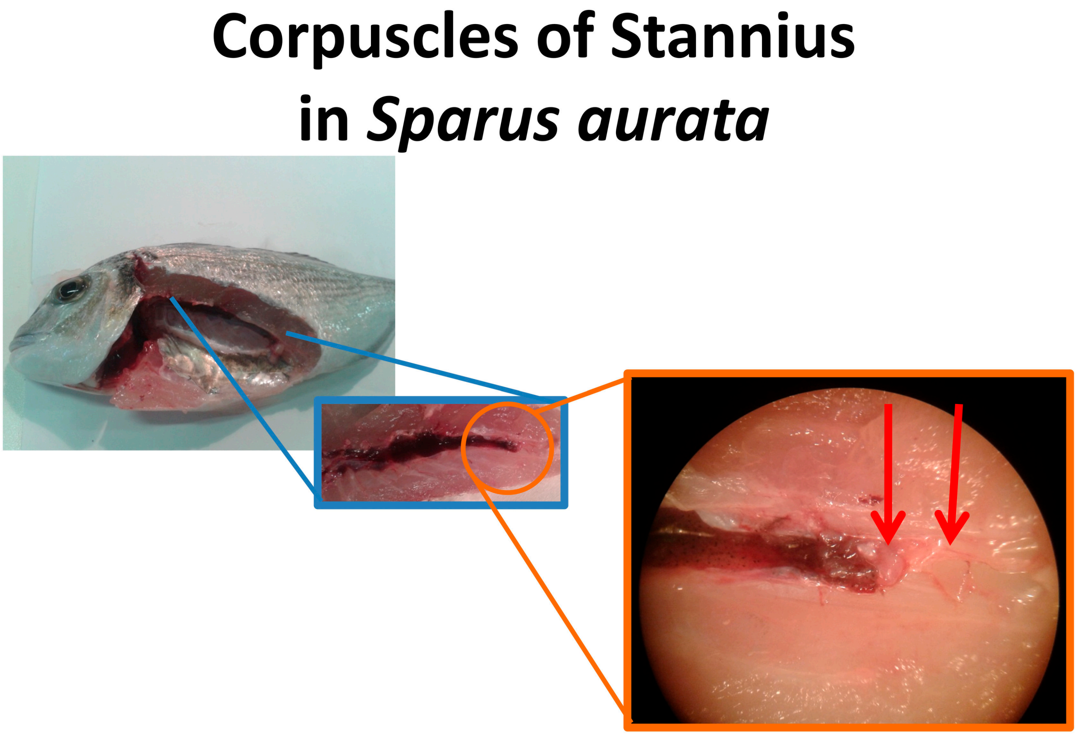

Figure S2. ELISA displacement curves. Displacement curves of purified STC standards (standard curve, black dots) and serial dilutions of plasma (white dots) and corpuscles of Stannius (CS) homogenates (black triangles) from gilthead seabream (*Sparus aurata*). Each point is the mean of four determinations. Bi, specifically bound tracer; Bo, maximum bound tracer.

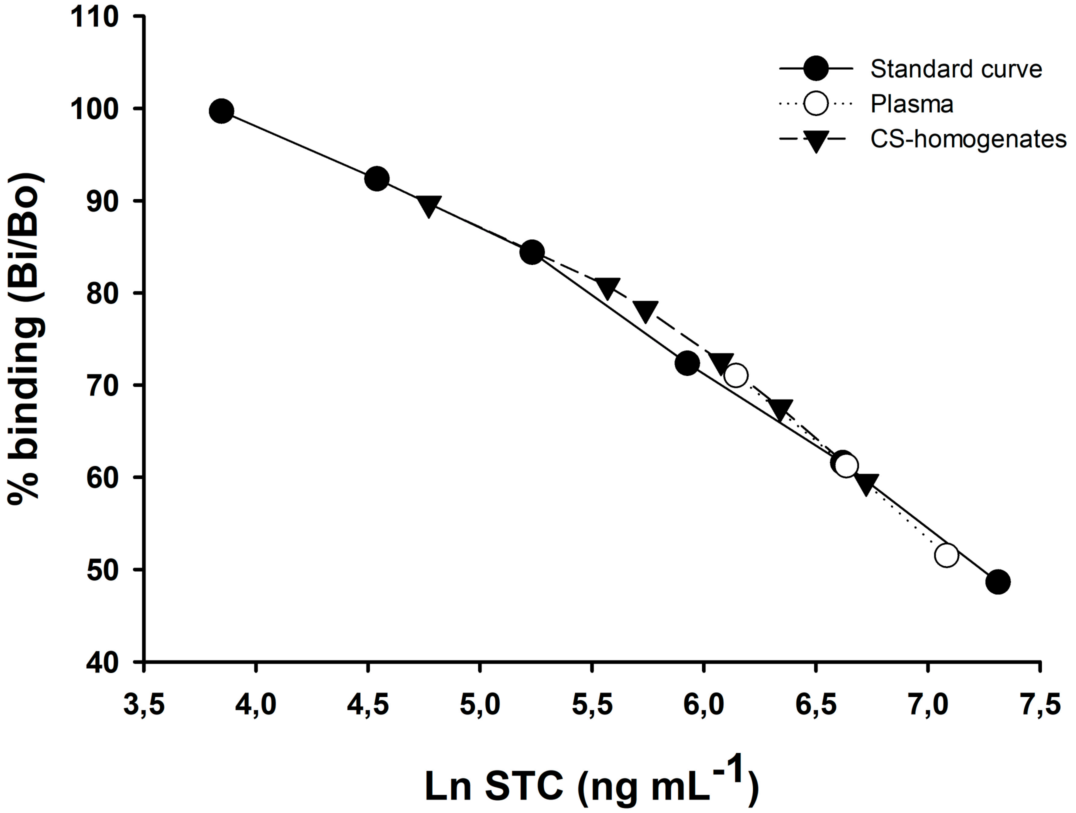

Figure S3. CS incubation and STC secretion along time. Secreted STC from *ex vivo* incubated corpuscles of Stannius in the absence (control) or presence of 0.05% DMSO (DMSO). Incubation times were 3, 6 and 24 hours. Values indicate mean ± SEM (n = 4). Different letters indicate significant differences between groups (two-way ANOVA followed by a Tukey´s post-hoc test, $p < 0.05$).

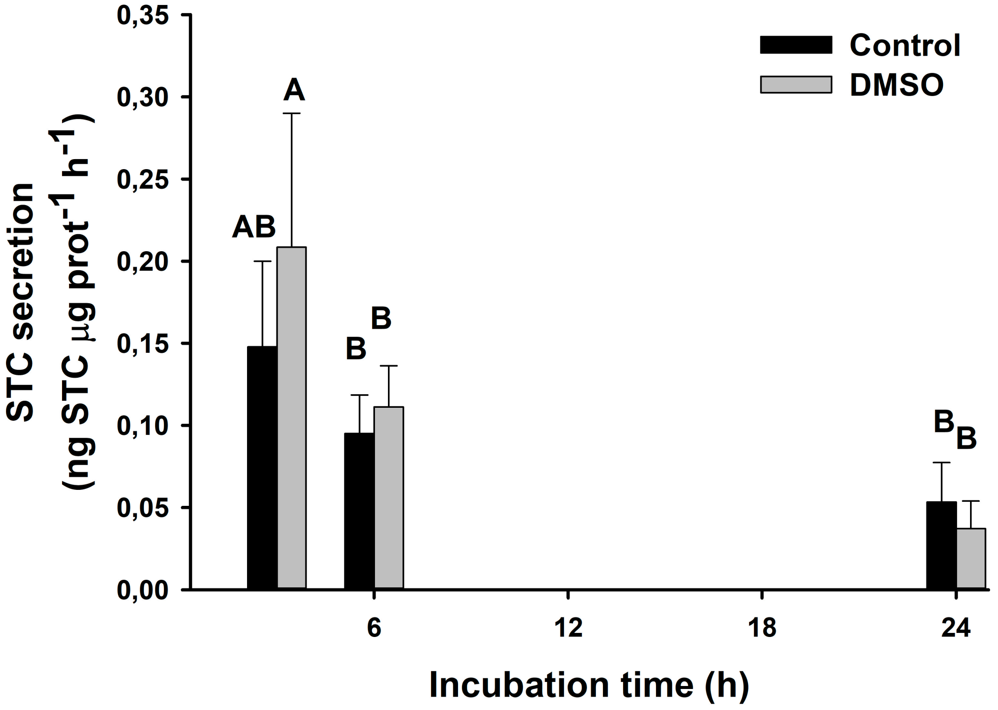

Supplement: Supplementary file 1 [file biology-11-00863-s001.zip › biology-1761873-supplementary.pdf]
